# The unique association between serum 25-hydroxyvitamin D concentrations and blood lipid profiles in agriculture, forestry, and fishing occupations: Insights from NHANES 2001–2014

**DOI:** 10.1371/journal.pone.0297873

**Published:** 2024-02-27

**Authors:** Baoshan Zhang, Xibin Dong

**Affiliations:** 1 Key Laboratory of Sustainable Forest Management and Environmental Microorganism Engineering of Heilongjiang Province, Northeast Forestry University, Harbin, China; 2 Faculty of Forestry and Environmental Management, University of New Brunswick, Fredericton, Canada; Muhimbili University of Health and Allied Sciences School of Medicine, UNITED REPUBLIC OF TANZANIA

## Abstract

**Background:**

The relationship of serum 25(OH)D levels and hyperlipidemia has not been explored in the Agriculture, Forestry, and Fishing (AFF) occupation. We aimed to explore the impact of serum 25(OH)D levels on lipid profiles in AFF workers, traffic drivers, and miners.

**Methods:**

Data from 3937 adults aged 18–65 years old with completed information were obtained from the National Health and Examination Survey from 2001 to 2014. Multivariate linear regression models were used to examine the associations between serum 25(OH)D concentrations and triglycerides (TG), total cholesterol (TC), low-density lipoprotein cholesterol (LDL-C), high-density lipoprotein cholesterol (HDL-C) and HDL-C/LDL-C ratio. Subgroup analyses for AFF workers considered age, sex, BMI, work activity, months worked, and alcohol consumption. Non-linear relationships were explored using curve fitting.

**Results:**

Serum 25(OH)D levels differed between groups (AFF: 60.0 ± 21.3 nmol/L, drivers: 56.6 ± 22.2 nmol/L, miners: 62.8 ± 22.3 nmol/L). Subgroup analysis of the AFF group showed that participants with serum 25(OH)D ≥50 nmol/L, females, and BMI <30 kg/m^2^ demonstrated improved HDL-C levels correlating with higher serum 25(OH)D. Serum 25(OH)D in AFF workers had a reversed U-shaped relationship with TG and TC, and a U-shaped relationship with HDL-C, with HDL-C, with inflection points at 49.5 nmol/L for TG and TC, and 32.6 nmol/L for HDL-C.

**Conclusions:**

Serum 25(OH)D levels are associated with lipid profiles, and the relationship varies among occupational groups. AFF workers, facing unique occupational challenges, may benefit from maintaining adequate serum 25(OH)D levels to mitigate adverse lipid profiles and reduce cardiovascular risk.

## Introduction

Hyperlipidemia, characterized by dysregulation of triglycerides (TG), total 3 cholesterol (TC), low-density lipoprotein cholesterol (LDL-C) and high-density lipoprotein cholesterol (HDL-C), is a significant risk factor for atherosclerosis, cardiovascular and immune-related diseases [1–3]. High TG levels, reduced HDL-C, and atherogenic small dense LDL (sdLDL) collectively contribute to the metabolic insulin resistance syndrome, which exhibits a nearly linear association with cardiovascular disease (CVD) risk [4].

25-hydroxyvitamin D [25(OH)D]-the primary storage form of vitamin D, plays a crucial role in regulating calcium and phosphate metabolism, promoting bone health, supporting immune function [5]. Moreover, emerging evidence suggests that serum 25(OH)D may also contribute to reducing the risk of mortality, particularly among individuals with hypertension and type 2 diabetes [6]. Sun exposure and oral intake are the two main sources of serum 25(OH)D, including vitamin D supplementation and dietary intake [7]. The relationship between serum 25(OH)D concentrations and hyperlipidemia has been investigated in several studies with some indicating a negative association between serum 25(OH)D levels and lipid profiles such as TC, TG, and LDL-C [8–10], while others have not found a clear association [11, 12]. These discrepancies may be attributed to differences in study design, sample characteristics, and the presence of other confounding factors. Additionally, the relationship between vitamin D and lipid metabolism is complex and influenced by various factors, including sunlight exposure, diet, physical activity, genetic factors, and other environmental factors [13]. In the United States, workers in the Agriculture, Forestry, and Fishing (AFF) occupations are commonly referred to as the labor force within the agricultural sector [14]. In 2004, the AFF sector employed around 2.1 million workers, solidifying its position as one of the United States’ largest economic sectors [15]. Despite numerous studies indicating that individuals working in AFF occupations are prone to diseases associated with exposure to sunlight and pesticides due to the nature of their work [16–18], no reports have yet explored the specific relationship between serum 25(OH)D concentrations and hyperlipidemia within the AFF population.

Given the conflicting findings regarding the association between serum 25(OH)D concentrations and hyperlipidemia, as well as the complexity of lipid metabolism and the unique characteristics of AFF occupations, our study aims to explore the effects of increasing serum 25(OH)D levels on changes in blood lipid profiles based on a large and representative US general population from the National Health and Nutrition Examination Survey (NHANES) from 2001 to 2014. Considering that physical activity and sun exposure may be pivotal factors influencing the relationship between serum 25(OH)D concentrations and lipid profiles in AFF, we selected two control groups within AFF: traffic drivers and miners: regarding physical activity, the traffic drivers represent a subgroup of workers characterized by non-vigorous physical activity during work and limited sun exposure, whereas miners constitute a subgroup engaged in vigorous physical activity at work but also lacking significant sun exposure.

## Materials and methods

### Study population

**Fig 1:**
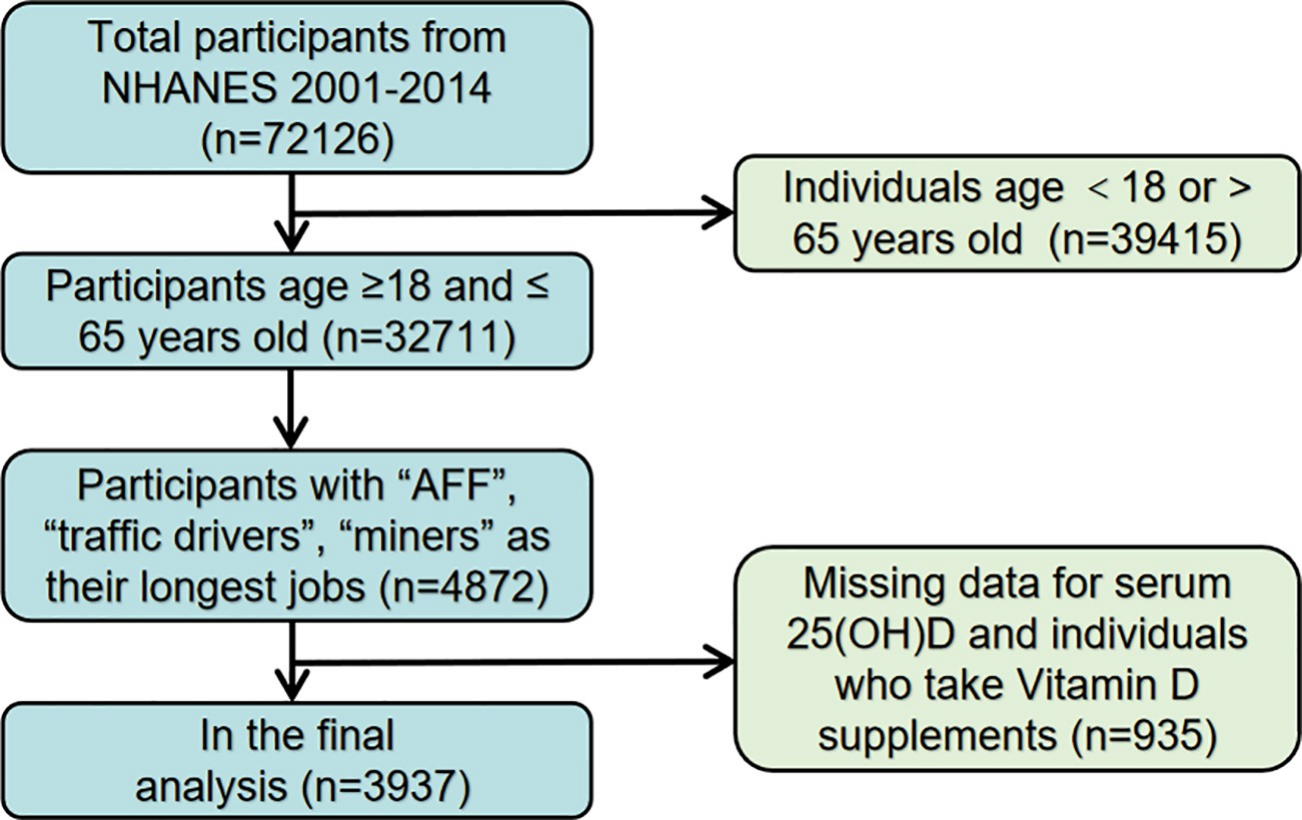
Flow chat of sample selection from the NHANES 2001–2014.

This study employed a cross-sectional analysis using data from seven cycles (2001–2014) of the National Health and Nutrition Examination Survey (NHANES), a vital survey conducted collaboratively by the Centers for Disease Control and Prevention (CDC) and the National Center for Health Statistics (NCHS) in the US. A total of 32,711 participants aged 18 to 65 years were initially recruited. From these, we selected individuals based on the ’Kind of work you have done the longest’ category in the NHANES occupation section. We then identified their specific job classifications using the survey’s supplementary tables. For our analysis, we focused on 4,872 individuals who had AFF, traffic driving, and mining as their longest-held jobs. After excluding participants with incomplete serum 25(OH)D concentrations data (n = 935), our analysis included a final sample of 3937 participants with complete data on both serum 25(OH)D concentrations and lipid profiles. A schematic representation of the sample selection process is depicted in (Fig 1).

The NHANES study obtained ethical clearance from the Research Ethics Review Board of the National Center for Health Statistics and the documented consent was obtained from participants (Protocol #98–12, #2005–06, #2011–17). Written informed consent was obtained from all participants. Further information can be found at https://www.cdc.gov/nchs/nhanes/irba98.htm.

### Variables

#### Serum 25(OH)D concentrations

From 2001 to 2006, serum 25(OH)D concentrations were measured using the radio immunoassay (RIA) method. Subsequently, from 2007 to 2014, a standardized liquid chromatography-tandem mass spectrometry (LC-MS/MS) method was employed. According to the NHANES recommendations, serum 25(OH)D concentrations data from 2001 to 2006 have been converted by using regression to equivalent 25(OH)D measurements from a standardized liquid chromatography-tandem mass spectrometry (LC-MS/MS) method. Detailed descriptions of the laboratory methods and protocols can be found on the NHANES website (http://www.cdc.gov/nchs/nhanes/).

In our analysis, we categorized serum 25(OH)D concentrations into two groups based on the definitions of vitamin D sufficiency and deficiency according to the USA Institute of Medicine (IOM): vitamin D sufficiency with ≥ 50 nmol/L and < 50 nmol/L as vitamin D deficiency [19, 20].

#### Outcomes

This study focused on 5 outcome variables: TC, TG, HDL-C, LDL-C, obtained from NHANES laboratory data, along with the HDL-C to LDL-C ratio. Timed-endpoint methods were used to measure the concentrations of these four lipids [21].

#### Other variables

The categorical variables were: sex, race, education level, marital status, vigorous work activity (Activities that require hard physical effort and cause large increases in breathing or heart rate. The participants were asked “Does your work involve vigorous-intensity activity that causes large increases in breathing or heart rate like carrying or lifting heavy loads, digging or construction work for at least 10 minutes continuously?”), hypertension (whether you have been informed by a doctor about high blood pressure), diabetes (whether you have been informed by a doctor about having diabetes), hyperlipidemia (whether you have been informed by a doctor about having high cholesterol), smoking status (whether you have smoked at least 100 cigarettes in your lifetime), heavy alcohol consumption (Ever have 4/5 or more drinks every day). Furthermore, race was classified as Hispanic, non-Hispanic White, non-Hispanic Black, or others. Each race was included separately as a dummy variable, e.g., “Hispanic”- yes/no, “Non-Hispanic White”- yes/no, “Non-Hispanic Black”- yes/no, “Others”-yes/no.

Additionally, we included several continuous covariates, including age, body mass index (BMI), arm circumference, waist circumference, energy intake (NHANES participants underwent two 24-hour dietary recall interviews, with the first conducted in-person at the Mobile Examination Center (MEC) and the second via telephone 3 to 10 days later. We utilized the average of these two sets of data), other metabolic indexes (serum glucose, glycohemoglobin, blood urea nitrogen, uric acid and creatinine). Detailed information on serum 25(OH)D concentrations, TC, TG, LDL-C, HDL-C, and other covariates can be found on the NHANES website, which is publicly accessible (http://www.cdc.gov/nchs/nhanes/). Besides, we also divided BMI into three groups based on the official (WHO) cut-offs for nutritional status: < 25 kg/m^2^ (normal weight and underweight), 25–30 kg/m^2^ (overweight), and ≥ 30 kg/m^2^ (obese) [22], as well as divided the lipid profiles into two groups: TG < or ≥ 1.7 mmol/L, TC < or ≥5.2 mmol/L, LDL-C < or ≥ 3.4 mmol/L, HDL ≤ or > 1.0 mmol/L for men and 1.3 mmol/L for women [23], and ≤ or > 0.4 mmol/L for HDL-C/LDL-C ratio [24, 25].

### Statistical analysis

The analysis was conducted following the recommendations of the NCHS. Categorical variables were presented as numbers with percentages, while continuous variables were expressed as means ± standard deviations (SDs). The normality of the distribution of continuous data was tested by using the Kolmogorov–Smirnov test. Differences among the three occupation groups were assessed using chi-square tests for categorical variables and by Student T test or Mann-Whitney test, depending on the parametric or non-parametric distribution of continuous data.

To explore the relationship between serum 25(OH)D concentrations and lipid profiles, multivariable regression models were constructed: Model 1 (unadjusted), Model 2 (adjusted for sex, age, each particular race, smoking, heavy alcohol consumption, diabetes, hypertension, vigorous physical activity, education level, and marital status), and Model 3 (additionally adjusted for BMI). Besides, the analyses were repeated with the inclusion of energy intake as a covariate in Models 2 and 3.

In addition, in the AFF occupation group, stratified analyses were conducted based on serum 25(OH)D concentrations (< 50 nmol/L or ≥ 50 nmol/L), sex, age (< 40 years old or ≥ 40 years old), BMI (< 30 kg/m^2^ or ≥ 30 kg/m^2^), vigorous work activity (yes or no), work duration (< 16.7 years or ≥ 16.7 years, see below) and heavy alcohol consumption (yes or no). Additionally, we performed subgroup analysis stratified by age, sex, BMI, vigorous work activity duration of working and heavy alcohol in AFF occupation, which aimed to provide further insights into the potential variations in lipid profiles concerning serum 25(OH)D concentrations among the AFF population.

Generalized additive models and smoothed curve fitting techniques were then employed to detect any nonlinear relationships between serum 25(OH)D concentrations and lipid profiles in the three distinct occupational groups, using a recursive algorithm for calculating infection. The analyses were adjusted by sex, age, each particular race, BMI, smoking, heavy alcohol, diabetes, hypertension, vigorous physical activity, education level, and marital status (i.e., from Model 3).

All statistical analyses were performed using the R software package (http://www.Rproject.org) and Empower Stats (http://www.empowerstats.com). Statistical significance was determined at a p-value threshold of less than 0.05.

## Results

### Description of participant characteristics

Detailed baseline characteristics of all participants were presented in Table 1. The three occupational groups did not differ regarding age (about 40–41 years old). In all of them, the majority were males, but the most in the miners (above 90%) and the least in the AFF occupations (about three-quarters). Among the AFF group, about 60% were Hispanic and about 30% were non-Hispanic White, while in the traffic drivers and miners, the non-Hispanic White and non-Hispanic Black ethnicity was much more represented.

**Table 1:** Baseline characteristics of 3937 participants according to occupation.

Characteristics	AFF	Traffic drivers	Miners	P-values		
				AFF vs. drivers	AFF vs. miners	divers vs. miners
Participants (No.)	625	1604	1708	-	-	-
Serum 25(OH)D (nmol/L)	60.0 ± 21.3	56.6 ± 22.2	62.8 ± 22.3	0.001**	0.006**	<0.001***
Working months	142.2 ± 125.7	122.8 ± 114.9	159.8 ± 129.8	0.010*	0.030*	<0.001***
TG (mmol/L)	2.0 ± 1.6	1.8 ± 1.6	2.0 ± 1.9	0.104	0.654	0.008**
TC (mmol/L)	5.1 ± 1.1	5.0 ± 1.2	5.1 ± 1.1	0.072	0.815	0.029*
LDL-C (mmol/L)	3.1 ± 0.9	3.0 ± 1.0	3.0 ± 0.9	0.165	0.339	0.495
HDL-C (mmol/L)	1.2 ± 0.3	1.3 ± 0.4	1.3 ± 0.4	0.627	0.690	0.909
HDL/LDL ratio	0.5 ± 0.2	0.5 ± 0.3	0.5 ± 0.3	0.106	0.338	0.261
Age (years)	40.6 ± 14.9	41.4 ± 14.0	41.4 ± 13.5	0.254	0.208	0.915
Serum glucose (mmol/L)	5.7 ± 2.2	5.7 ± 2.3	5.6 ± 2.0	0.819	0.697	0.407
BMI (kg/m^2^)	28.6 ± 6.1	29.5 ± 7.1	28.3 ± 5.8	0.005**	0.285	<0.001***
Arm circumference (cm)	33.3 ± 4.5	34.5 ± 5.2	33.9 ± 4.3	<0.001***	0.004**	<0.001***
Waist circumference (cm)	98.1 ± 15.3	101.1 ± 17.6	98.3 ± 14.9	<0.001***	0.728	<0.001***
Creatinine (μmol/L)	74.5 ± 18.2	85.4 ± 43.9	82.2 ± 18.7	<0.001***	<0.001***	0.006**
Blood urea nitrogen (mmol/L)	4.5 ± 1.7	4.4 ± 1.7	4.4 ± 1.5	0.032*	0.115	0.322
Serum uric acid (μmol/L)	326.4 ± 83.6	344.3 ± 80.9	347.3 ± 77.3	<0.001***	<0.001***	0.266
Glycohemoglobin (%)	5.7 ± 1.2	5.7 ± 1.2	5.7 ± 1.1	0.643	0.424	0.086
Energy intake (kcal)	2315.6 ± 1040.5	2419.4 ± 1031.4	2592.6 ± 1096.6	0.039*	<0.001***	<0.001***
Serum 25(OH)D categories (%)				0.008**	0.005**	<0.001***
< 50 nmol/L	221 (35.4%)	665 (41.5%)	500 (29.3%)			
≥ 50 nmol/L	404 (64.6%)	939 (58.5%)	1208 (70.7%)			
BMI categories (%)				0.021*	0.339	<0.001***
< 25 kg/m^2^	166 (27.2%)	421 (26.6%)	510 (30.2%)			
25–30 kg/m^2^	242 (39.6%)	543 (34.3%)	627 (37.1%)			
≥ 30 kg/m^2^	203 (33.2%)	621 (39.2%)	553 (32.7%)			
TG categories (%)				0.246	0.827	0.062
< 1.7 mmol/L	359 (57.8%)	965 (60.5%)	973 (57.3%)			
≥ 1.7 mmol/L	262 (42.2%)	630 (39.5%)	725 (42.7%)			
TC categories (%)				0.043*	0.960	0.005**
< 5.2 mmol/L	342 (54.9%)	953 (59.6%)	934 (54.8%)			
≥ 5.2 mmol/L	281 (45.1%)	646 (40.4%)	771 (45.2%)			
LDL-C categories (%)				0.191	0.560	0.318
< 3.4 mmol/L	186 (65.3%)	504 (69.5%)	554 (67.2%)			
≥ 3.4 mmol/L	99 (34.7%)	221 (30.5%)	271 (32.8%)			
HDL-C categories (%)				0.140	<0.001***	0.012*
≤ 1.0 or 1.3 mmol/L	207 (33.2%)	479 (30.0%)	444 (26.0%)			
> 1.0 or 1.3 mmol/L	417 (66.8%)	1120 (70.0%)	1261 (74.0%)			
HDL/LDL ratio categories (%)				0.854	0.646	0.714
≤ 0.4 mmol/L	133 (46.7%)	343 (47.3%)	398 (48.2%)			
> 0.4 mmol/L	152 (53.3%)	382 (52.7%)	427 (51.8%)			
Sex (%)				<0.001***	<0.001***	<0.001***
Males	453 (72.5%)	1296 (80.8%)	1580 (92.5%)			
Females	172 (27.5%)	308 (19.2%)	128 (7.5%)			
Race (%)						
Hispanic				<0.001***	<0.001***	<0.001***
Yes	377 (60.3%)	450 (28.1%)	650 (38.1%)			
No	248 (39.7%)	1154 (71.9%)	1058 (61.9%)			
Non-Hispanic White				0.013*	<0.001***	<0.001***
Yes	192 (30.7%)	582 (36.3%)	720 (42.2%)			
No	433 (69.3%)	1022 (63.7%)	988 (57.8%)			
Non-Hispanic Black				<0.001***	<0.001***	<0.001***
Yes	38 (6.1%)	486 (30.3%)	271 (15.9%)			
No	587 (93.9%)	1118 (69.7%)	1437 (84.1%)			
Others				0.013*	0.234	0.049*
Yes	18 (2.9%)	86 (5.4%)	67 (3.9%)			
No	607 (97.1%)	1518 (94.6%)	1641 (96.1%)			
Hypertension (%)				<0.001***	0.008**	0.005**
Yes	119 (19.7%)	469 (29.3%)	426 (25.0%)			
No	485 (80.3%)	1130 (70.7%)	1278 (75.0%)			
Hyperlipidemia (%)				0.558	0.506	0.058
Yes	108 (35.5%)	405 (37.4%)	368 (33.5%)			
No	196 (64.5%)	679 (62.6%)	731 (66.5%)			
Diabetes (%)				0.015*	0.795	0.002**
Yes	56 (9.0%)	203 (12.7%)	159 (9.3%)			
No	568 (91.0%)	1401 (87.3%)	1546 (90.7%)			
Smoking status (%)				<0.001***	<0.001***	<0.001***
Yes	246 (43.7%)	819 (54.2%)	1067 (65.1%)			
No	317 (56.3%)	693 (45.8%)	571 (34.9%)			
Heavy alcohol (%)				0.744	0.001**	<0.001***
Yes	107 (24.3%)	322 (25.1%)	475 (32.4%)			
No	333 (75.7%)	961 (74.9%)	993 (67.6%)			
Vigorous work activity (%)				0.435	<0.001***	<0.001***
Yes	198 (31.7%)	481 (30.0%)	744 (43.6%)			
No	427 (68.3%)	1123 (70.0%)	963 (56.4%)			
Education level (%)				<0.001***	<0.001***	<0.001***
Less than high school	373 (60.8%)	529 (33.0%)	719 (42.2%)			
High school and above	240 (39.2%)	1073 (67.0%)	984 (57.8%)			
Marital status (%)				0.010*	0.160	0.106
Married or with a partner	405 (66.3%)	938 (60.3%)	1048 (63.1%)			
Others	206 (33.7%)	617 (39.7%)	613 (36.9%)			

**Notes:** Mean ± SD for continuous variables and between groups P values were calculated by Student T test or Mann-Whitney test, depending on the parametric or non-parametric distribution of continuous data.

* P <0.05;

** P <0.01;

*** P <0.001.

The average serum 25(OH)D concentrations in the AFF workers were 60.0 ± 21.3, and about two-thirds had vitamin D sufficient levels. Among these three occupational groups, miners exhibited the highest (62.8 ± 22.3 nmol/L), while traffic drivers had the lowest serum 25(OH)D concentrations values (56.6 ± 22.2 nmol/), which statistically differed from the AFF group. The differences in lipid profiles, glucose, and glycohemoglobin were mostly non-significant. In all three groups, there was a high proportion of the subjects with increased TG (about 40–43%), TC (40–45%), LDL-C (30–35%), and decreased HDL-C levels (26–33%) and HDL-C/LDL-C ratio (47–48%), with one-third of the subjects being previously diagnosed with dyslipidemia. In contrast, the AFF group had lower BMI and waist circumference compared to traffic drivers, and lower arm circumference, serum creatinine, uric acid, and energy intakes compared to both two other groups. In the AFF group and miners, the majority were overweight, while among drivers, the majority were obese. In all three groups, only about 30% or less were the normal weight subjects. The AFF subjects differed from both two other groups regarding the lower percentage of hypertension, lower educational level, and lower percentage of smokers, while from miners only differed by the lower percentage of vigorous work physical activity and heavy alcohol consumption (Table 1).

### Different relationships of serum 25(OH)D concentrations with lipid profiles in three occupations

We designed multivariate linear regression models to investigate the independent associations of serum 25(OH)D levels with lipid profiles. After conducting the univariate regression analyses (S1, S2 Tables in S1 Appendix), sex, age, each particular race, smoking, heavy alcohol consumption, diabetes, hypertension, vigorous physical activity, education level, and marital status were added as covariates in multivariate regression analyses (Model 2), while BMI was additionally added as a covariate in the fully adjusted model (Model 3) (Tables 2 and 3). Some of the variables from S1, S2 Tables in S1 Appendix were excluded from covariates due to the issue of multicollinearity, while energy intake was excluded due to high inter-day variability and weak correlation with lipid levels.

**Table 2:** The associations of serum 25(OH)D concentrations with lipid profiles in three occupations.

	Outcomes: (β (95% CI) P-value)				
	TG (mmol/L)	TC (mmol/L)	HDL-C (mmol/L)	LDL-C (mmol/L)	HDL-C/LDL-C
**Total**					
Model 1	-0.002 (-0.004, 0.001) 0.20	-0.000 (-0.002, 0.001) 0.90	-0.000 (-0.001, 0.000) 0.82	-0.000 (-0.002, 0.002) 0.85	-0.000 (-0.001, 0.000) 0.72
Model 2	-0.004 (-0.007, -0.001) < 0.01**	-0.001 (-0.003, 0.001) 0.37	0.001 (0.000, 0.001) 0.02*	-0.000 (-0.003, 0.002) 0.73	0.000 (-0.000, 0.001) 0.24
Model 3	-0.002 (-0.006, 0.001) 0.12	-0.001 (-0.003, 0.001) 0.56	0.000 (-0.001, 0.001) 0.78	-0.000 (-0.003, 0.002) 0.98	0.000 (-0.001, 0.001) 0.82
**AFF**					
Model 1	-0.003 (-0.009, 0.003) 0.28	-0.001 (-0.005, 0.003) 0.67	0.002 (0.000, 0.003) 0.01*	-0.001 (-0.006, 0.003) 0.53	0.001 (0.000, 0.002) 0.003**
Model 2	-0.002 (-0.010, 0.007) 0.68	0.000 (-0.006, 0.006) 0.99	0.002 (0.000, 0.004) 0.03*	0.000 (-0.006, 0.006) 0.97	0.001 (-0.000, 0.002) 0.08
Model 3	-0.001 (-0.009, 0.007) 0.84	0.001 (-0.005, 0.007) 0.80	0.001 (-0.001, 0.003) 0.20	0.001 (-0.005, 0.007) 0.79	0.001 (-0.001, 0.002) 0.23
**Traffic drivers**					
Model 1	0.001 (-0.003, 0.004) 0.71	0.001 (-0.002, 0.003) 0.51	-0.001 (-0.001, 0.000) 0.25	0.000 (-0.003, 0.003) 0.86	-0.000 (-0.001, 0.001) 0.36
Model 2	-0.003 (-0.008, 0.001) 0.14	0.000 (-0.003, 0.003) 0.93	0.001 (0.000, 0.002) 0.04*	-0.001 (-0.005, 0.003) 0.72	0.001 (-0.000, 0.002) 0.15
Model 3	-0.002 (-0.006, 0.002) 0.38	0.000 (-0.003, 0.004) 0.86	0.000 (-0.001, 0.001) 0.36	-0.001 (-0.005, 0.003) 0.74	0.001 (-0.000, 0.002) 0.23
**Miners**					
Model 1	-0.003 (-0.007, 0.001) 0.12	-0.001 (-0.003, 0.002) 0.55	-0.000 (-0.001, 0.001) 0.64	-0.000 (-0.003, 0.003) 0.94	-0.000 (-0.001, 0.000) 0.36
Model 2	-0.006 (-0.011, -0.000) 0.03*	-0.002 (-0.005, 0.001) 0.21	0.000 (-0.001, 0.001) 0.66	0.000 (-0.003, 0.004) 0.92	-0.001 (-0.002, 0.000) 0.26
Model 3	-0.003 (-0.008, 0.002) 0.23	-0.001 (-0.004, 0.002) 0.36	-0.001 (-0.002, 0.000) 0.20	0.001 (-0.003, 0.005) 0.60	-0.001 (-0.002, -0.000) 0.02*

Model 1: Non-adjusted

Model 2: Adjusted for sex, age, each particular race, smoking, heavy alcohol, diabetes, hypertension, vigorous physical activity, education level, and marital status.

Model 3: Adjusted for covariates in Model 2 with additionally BMI included.

* P <0.05,

** P <0.01.

**Table 3:** The stratified analyses of the associations of serum 25(OH)D concentrations with lipid profiles in AFF occupations (with fully adjusted model).

	Outcomes: (β (95% CI) P-value)				
	TG (mmol/L)	TC (mmol/L)	HDL-C (mmol/L)	LDL-C (mmol/L)	HDL-C/LDL-C
**Total**	-0.001 (-0.009, 0.007) 0.84	0.001 (-0.005, 0.007) 0.80	0.001 (-0.001, 0.003) 0.20	0.001 (-0.005, 0.007) 0.79	0.001 (-0.001, 0.002) 0.23
**Serum 25(OH)D group (nmol/L)**					
< 50	0.027 (-0.007, 0.061) 0.12	0.021 (-0.006, 0.047) 0.13	-0.005 (-0.013, 0.003) 0.19	-0.010 (-0.043, 0.023) 0.57	-0.000 (-0.006, 0.006) 0.96
≥ 50	-0.010 (-0.021, 0.001) 0.08	-0.004 (-0.013, 0.005) 0.39	0.003 (0.001, 0.005) < 0.01**	0.002 (-0.007, 0.010) 0.72	0.002 (-0.000, 0.004) 0.09
**Age (years)**					
< 40	0.000 (-0.014, 0.015) 0.97	0.005 (-0.006, 0.016) 0.34	0.002 (-0.001, 0.005) 0.25	-0.002 (-0.015, 0.010) 0.75	0.001 (-0.002, 0.004) 0.39
≥ 40	-0.002 (-0.011, 0.008) 0.75	-0.001 (-0.008, 0.006) 0.73	0.001 (-0.001, 0.003) 0.29	0.001 (-0.007, 0.008) 0.83	0.001 (-0.000, 0.002) 0.17
**Sex**					
Males	0.002 (-0.008, 0.012) 0.72	0.001 (-0.006, 0.008) 0.77	-0.000 (-0.002, 0.001) 0.64	0.002 (-0.006, 0.010) 0.61	0.000 (-0.001, 0.002) 0.66
Females	-0.007 (-0.019, 0.005) 0.28	-0.000 (-0.011, 0.010) 0.93	0.004 (0.001, 0.007) 0.02*	-0.001 (-0.012, 0.011) 0.87	0.002 (-0.001, 0.004) 0.23
**BMI (kg/m** ^**2**^ **)**					
< 30	-0.003 (-0.012, 0.007) 0.60	0.003 (-0.004, 0.010) 0.37	0.003 (0.001, 0.005) 0.01*	0.002 (-0.005, 0.009) 0.57	0.001 (-0.000, 0.003) 0.11
≥ 30	0.004 (-0.009, 0.018) 0.54	-0.003 (-0.015, 0.008) 0.56	-0.002 (-0.005, 0.000) 0.10	0.000 (-0.015, 0.016) 0.95	-0.001 (-0.003, 0.002) 0.65
**Vigorous work activity**					
Yes	0.001 (-0.012, 0.014) 0.90	-0.002 (-0.013, 0.009) 0.71	0.002 (-0.001, 0.005) 0.24	0.000 (-0.012, 0.012) 0.98	0.001 (-0.002, 0.004) 0.36
No	-0.001 (-0.011, 0.010) 0.91	0.002 (-0.005, 0.009) 0.64	0.001 (-0.002, 0.003) 0.63	0.001 (-0.007, 0.009) 0.86	0.000 (-0.001, 0.002) 0.59
**Working months**					
< 200	-0.002 (-0.017, 0.012) 0.76	-0.006 (-0.018, 0.006) 0.33	0.001 (-0.002, 0.004) 0.61	-0.009 (-0.023, 0.005) 0.21	0.001 (-0.002, 0.005) 0.37
≥ 200	0.002 (-0.019, 0.022) 0.87	0.000 (-0.016, 0.017) 0.97	-0.000 (-0.004, 0.004) 0.87	0.009 (-0.012, 0.029) 0.42	-0.000 (-0.003, 0.002) 0.76
**Heavy alcohol**					
Yes	0.005 (-0.014, 0.023) 0.62	0.005 (-0.007, 0.016) 0.46	0.001 (-0.003, 0.005) 0.54	-0.001 (-0.014, 0.013) 0.94	0.001 (-0.002, 0.004) 0.48
No	-0.002 (-0.011, 0.007) 0.67	0.000 (-0.007, 0.007) 0.96	0.001 (-0.001, 0.003) 0.18	0.001 (-0.006, 0.009) 0.73	0.001 (-0.000, 0.003) 0.13

Sex, age, each particular race, BMI, smoking, heavy alcohol, diabetes, hypertension, vigorous physical activity, education level, and marital status were adjusted except the variable itself. (continuous age BMI was adjusted in the age and BMI subgroup).

* P <0.05,

** P <0.01.

In the univariate analyses, no significant associations were found between serum 25(OH)D and lipid profiles, except positive associations with HDL-C and HDL-C/LDL-C ratio in the AFF group only. The further analyses with adjustments for multiple covariates (except BMI), increased the significance of the association of serum 25(OH)D with TG levels (negative) and with HDL-C levels (positive) in the total sample. Nevertheless, the stratified analyses revealed that the associations with TG levels were the most significant among miners, while with HDL-C levels were the most significant among the AFF occupations and traffic drivers. However, further adjustment for BMI annulated the significance of these associations, indicating that the associations were mostly mediated by the effect of BMI.

### Stratified analyses on the relationships of serum 25(OH)D with lipid profiles in the AFF group

We additionally made stratified analyses according to categories of vitamin D status, age, sex, nutritional status, heavy alcohol consumption, vigorous work physical activity, and duration of work engagement in AFF occupations. We focused on the duration of work engagement in AFF occupations, as our supplementary analyses indicated a connection with the lipid profiles of workers in these sectors (S1 Fig in S1 Appendix). The results are shown in Table 3 (referring to the fully adjusted regression model, Model 3) and in S3 Table in S1 Appendix (referring to the adjusted model without BMI as a covariate, Model 2). The subgroup analyses revealed:

*Vitamin D Status*: When compared to the group with serum 25(OH)D concentrations < 50 nmol/L, ≥ 50 nmol/L group exhibited improved lipid profiles (decreased TG, and increased HDL-C levels and HDL-C/LDL-C ratio). However, the statistical significance was achieved only for HDL-C levels in the fully adjusted model.

*Sex*: Females exhibited more beneficial HDL-C levels in response to serum 25(OH)D compared to males.

*BMI*: Only within the subgroup with BMI <30, serum 25(OH)D levels were positively correlated with HDL-C levels.

*Age, Vigorous Work Activity, Work Duration, and Heavy Alcohol Consumption*:

There were no significant associations in either category.

In summary, the statistical significance in the associations between vitamin D and lipid was achieved only for HDL-C levels in individuals with vitamin D sufficient status, females, and non-obese individuals in the fully adjusted model.

### Smoothed curve fitting and two-piecewise linear regression model

**Fig 2:**
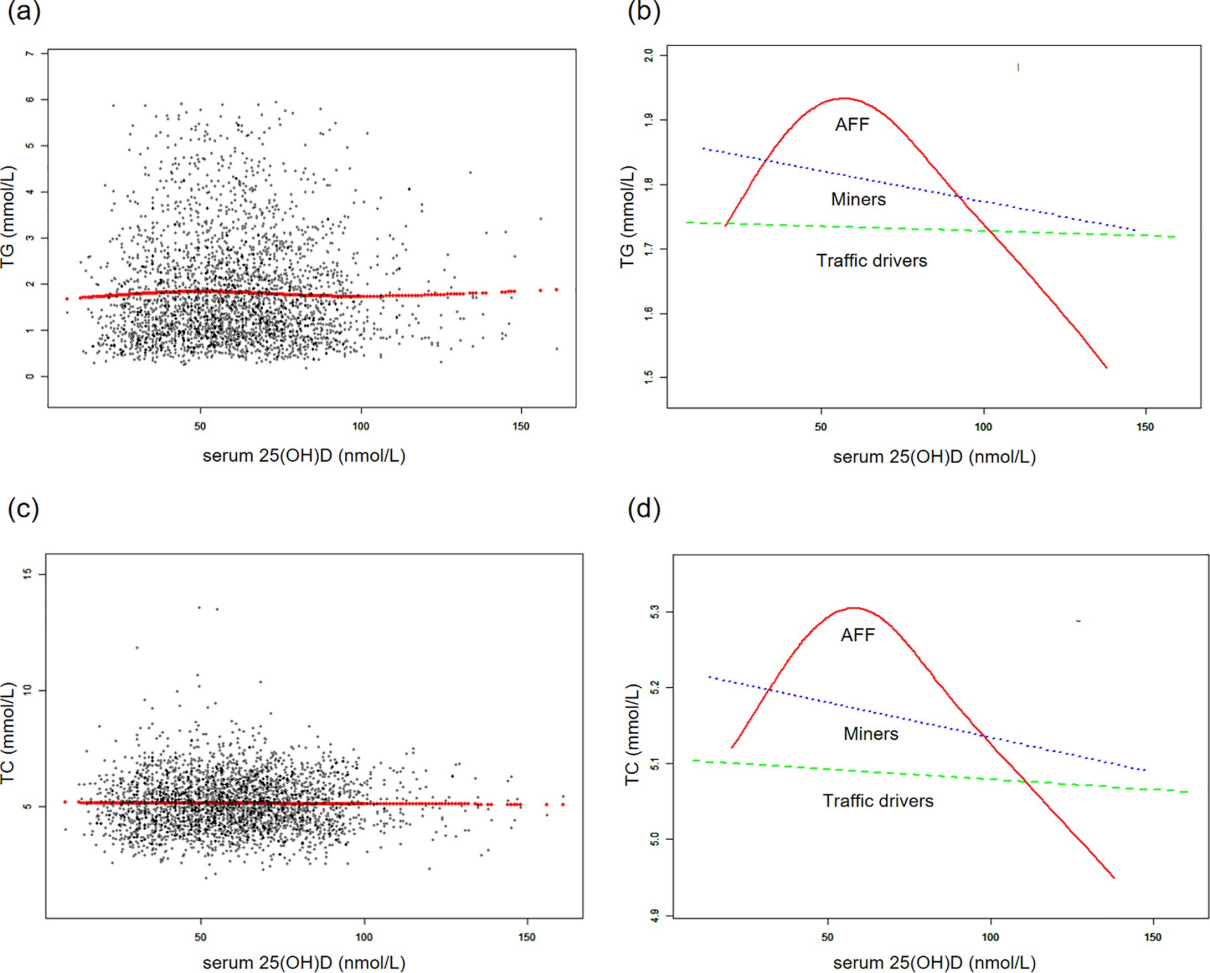
The associations between serum 25(OH)D concentrations and triglycerides, total cholesterol. **(a, c)** Each black point represents a sample and the red line represents the general trend of these samples. **(b, d)** Associations of serum 25(OH)D concentrations with triglycerides, total cholesterol stratified by occupations. Sex, age, each particular race, BMI, smoking, heavy alcohol, diabetes, hypertension, vigorous physical activity, education level, and marital status were adjusted.

**Fig 3:**
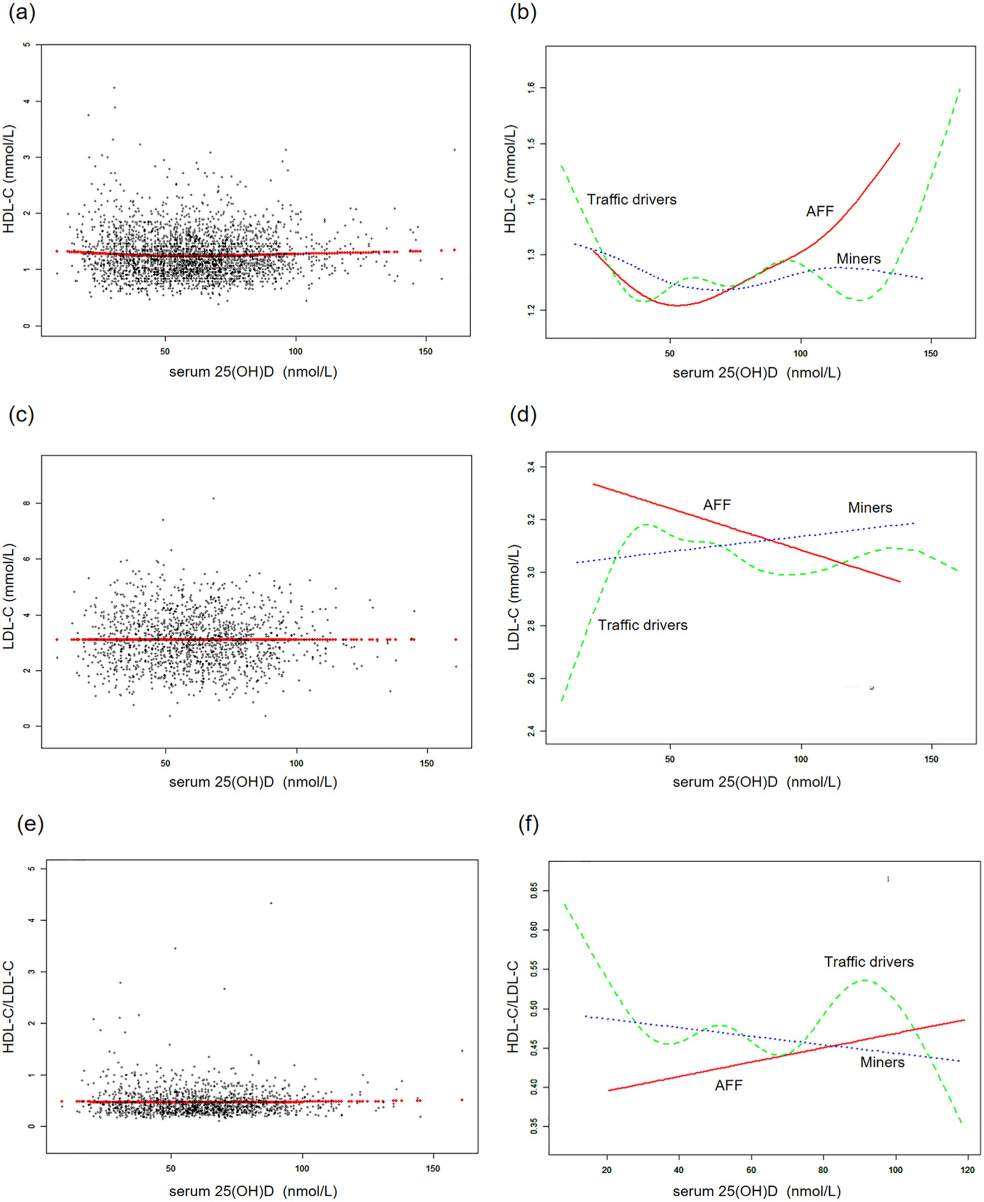
The associations between serum 25(OH)D concentrations and LDL cholesterol, LDL cholesterol, HDL-C/LDL-C. **(a, c, e)** Each black point represents a sample and the red line represents the general trend of these samples. **(b, d, f)** Associations of serum 25(OH)D concentrations with LDL cholesterol, HDL cholesterol and HDL-C/LDL/C stratified by occupations. Sex, age, each particular race, BMI, smoking, heavy alcohol, diabetes, hypertension, vigorous physical activity, education level, and marital status were adjusted.

Additionally, generalized additive models and smoothed curve fitting revealed different trends among the three occupational groups. Notably, in the AFF group, serum 25(OH)D concentrations exhibited a reversed U-shaped relationship with TG and TC, a U-shaped relationship with HDL-C, while they showed linear relationships with LDL-C and HDL-C/LDL-C (Figs 2 and 3). Further threshold analysis revealed that the inflection points for TG and TC were both at 49.5 nmol/L serum 25(OH)D, and for HDL-C, it was 32.6 nmol/L. However, there was no statistically significant linear or nonlinear relationship observed between LDL-C and the HDL-C/LDL-C ratio and serum 25(OH)D concentrations (Table 4). The results without BMI adjustment were shown in S2, S3 Figs in S1 Appendix, along with Table 4.

**Table 4:** Threshold effect analysis of serum 25(OH)D concentrations on lipid profiles.

	Adjusted HR (95% CI) P-value
**TG**	
Fitting by the standard linear model	-0.001 (-0.009, 0.007) 0.84
Fitting by the two-piecewise linear model	
Infection point	49.5
serum 25(OH)D < Infection point	0.028 (0.003, 0.053) 0.03*
serum 25(OH)D≥ Infection point	-0.008 (-0.018, 0.002) 0.11
P for Log-likelihood ratio	0.02*
**TC**	
Fitting by the standard linear model	0.001 (-0.006, 0.007) 0.87
Fitting by the two-piecewise linear model	
Infection point	49.5
serum 25(OH)D < Infection point	0.019 (0.001, 0.038) 0.04*
serum 25(OH)D ≥ Infection point	-0.005 (-0.013, 0.003) 0.23
P for Log-likelihood ratio	0.03*
**HDL-C**	
Fitting by the standard linear model	0.001 (-0.001, 0.003) 0.20
Fitting by the two-piecewise linear model	
Infection point	32.6
serum 25(OH)D < Infection point	-0.030 (-0.050, -0.010) < 0.01**
serum 25(OH)D ≥ Infection point	0.002 (0.000, 0.004) 0.03*
P for Log-likelihood ratio	< 0.01**
**LDL-C**	
Fitting by the standard linear model	0.001 (-0.005, 0.007) 0.79
Fitting by the two-piecewise linear model	
Infection point	58.7
serum 25(OH)D < Infection point	-0.004 (-0.018, 0.011) 0.63
serum 25(OH)D ≥ Infection point	0.003 (-0.006, 0.012) 0.52
P for Log-likelihood ratio	0.49
**HDL-C/LDL-C**	
Fitting by the standard linear model	0.001 (-0.001, 0.002) 0.23
Fitting by the two-piecewise linear model	
Infection point	102
serum 25(OH)D< Infection point	0.000 (-0.001, 0.002) 0.76
serum 25(OH)D ≥ Infection point	0.003 (-0.001, 0.007) 0.14
P for Log-likelihood ratio	0.24

Sex, age, each particular race, BMI, smoking, heavy alcohol, diabetes, hypertension, vigorous physical activity, education level, and marital status were adjusted.

* P <0.05,

** P <0.01.

## Discussion

This study is among the very rare ones that examined the correlation between serum 25(OH)D levels and lipid profiles within a population engaged in agriculture, forestry, and fishing professions. The associations of serum 25(OH)D concentrations with lipid profiles differed significantly among the three groups, highlighting the unique non-linear pattern observed in the AFF group when compared to both traffic workers and miners.

Our findings revealed that the average serum 25(OH)D concentrations in the AFF group were intermediate between those of traffic drivers (lower) and miners (higher), and fell into the vitamin D sufficiency range. About two-thirds of the subjects in the AFF group were vitamin D sufficient, while one-third were vitamin D deficient, which indicates that vitamin D deficiency is still present within the AFF sector. At the same time, all three professional groups showed pretty similar lipid profiles, with a high proportion of the subjects with increased TG, TC, LDL-C, and decreased HDL-C levels and HDL-C/LDL-C ratio. This was in agreement with the findings that in all three groups, the average BMI was in the range of overweight and that about 70% of the subjects in each group were overweight or obese, with slightly higher numbers among the traffic drivers. Moreover, this study found significantly lower serum creatinine and uric acid levels in the AFF group compared to two other groups (Table 1). This could indicate higher physical activity and healthier diets in the AFF group, potentially leading to better metabolic health [26]. Regarding serum glucose, no significant difference was noted among the three groups, with levels consistently within 5.7 ± 2.2 mmol/L. This implies that occupational type might not significantly influence blood sugar control. Notably, the AFF group’s lower BMI and waist circumference might be crucial in determining their metabolic markers. Generally, lower BMI and waist circumference correlate with improved metabolic health, likely explaining the AFF group’s lower creatinine and uric acid levels [27]. Although serum glucose levels were similar across all groups, the AFF group’s lower creatinine and uric acid levels may indicate better kidney function and metabolic status [28, 29].

Even though we have not found linear associations of serum 25(OH)D concentrations with serum lipid levels in the total sample and specific professions, the subgroup analyses within the AFF group indicated that vitamin D was linearly associated with better HDL-C levels in vitamin D sufficient individuals, females, and those without obesity. Besides, we found non-linear associations in the whole AFF group, indicating the inverse U-shaped pattern for the associations with TG and TC, and U-shaped pattern for HDL-C, with the inclination points about 50 nmol/L (for TG and TC) and 33 nmol/L (for HDL-C). However, the graphical presentation shows that 50 nmol/L could be also the inclination point for HDL-C.

The values of 50 nmol/L for the serum 25(OH)D concentrations are the official cut-off between vitamin D deficiency/insufficiency and vitamin D sufficiency according to the IOM, while according to the Endocrine Society, this is the border between vitamin D deficiency and vitamin D insufficiency [30, 31]. According to the IOM, the level of 30 nmol/L is the cut-off for severe deficiency [30, 31]. The official cut-offs for the serum 25(OH)D concentrations are related to the parathyroid hormone (PTH) concentration plateau at vitamin D levels of 50 nmol/L [30–32]. Of interest, PTH concentrations were associated with metabolic syndrome and lipid levels in numerous studies, particularly with TG and HDL-C levels, even independently of the BMI, with possibly sexually dimorphic associations [33–39]. It is possible that vitamin D deficiency, leading to high levels of PTH can negatively influence the metabolism of lipids. PTH can influence lipid levels through its effects on calcium and activation of vitamin D in the kidney, but it can have some more direct effects on insulin resistance, adipose tissue metabolism, lipolysis, and obesity development, as well as lipid metabolism in the liver [34–36]. Vitamin D, in contrast, can itself have effects on adipose tissue metabolism, differentiation, and adipogenesis, lipolysis, adipokines’ secretion, oxidative phosphorylation and insulin sensitivity in various tissues (influencing insulin signaling, glucose transporter, and mitochondrial function), lipid clearance in fat tissue and the muscle, lipid metabolism in the liver (the lipid particle components’ production, assembly, storage, and secretion), insulin secretion in the pancreas, other hormones secretion and action (sex hormones, glucocorticoids, mineralocorticoids, and renin-angiotensin-aldosterone system -RAAS), oxidative stress, and inflammation, and though all that ways modulate lipid metabolism, not only through its indirect effects on calcium and PTH [40–45]. In fact, all those direct and indirect mechanisms can be included [40, 46]. For instance, some of the mechanisms could be mediated through the association of vitamin D and PTH with obesity and BMI, but some independent associations can exist, as shown by our linear and two-piecewise regression models, in which some of the associations were lost after the additional adjustment for BMI, but some remained. In accordance, previous studies on mouse and human hepatoma cells indicated that the activation of the VDR (Vitamin D Receptor) inhibited the expression of FXR (Farnesoid X Receptor). This inhibition, in turn, led to the suppression of CYP7A1 (a cholesterol 7α-hydroxylase) expression, ultimately resulting in reduced cholesterol levels [41–43].

Our findings are in accordance with the previous studies in other populations [33, 40, 46–50]. However, the underlying mechanisms of the found associations are still unclear, and more research is warranted. Particularly, it is not clear why there are positive associations with the adverse lipid levels for vitamin D levels lower than the inclination points and why there is no influence on the LDL-C levels, while total TC levels are influenced. One of the possible explanations for the latter is that very low-density lipoprotein cholesterol (VLDL-C) levels, which are associated with TG levels, are also part of the TC levels, and that probably this fraction of TC is the most influenced by vitamin D. Of note is also that in the NHANES surveys the LDL-C values were not directly measured but were calculated according to the Friedewald equation, and this equation is not appropriate for the subjects with TG levels above 4.5 mmol/L [51].

Even though we observed quite different associations between serum 25(OH)D concentrations and lipid levels in the three occupational groups, we cannot give an explanation for the observed differences. One explanation is that the groups were not completely matched for demographic and lifestyle factors, and there is enough evidence that sex, ethnicity, lifestyle, and environmental factors (BMI, physical activity, dietary habits, alcohol consumption, smoking, sun exposure, and environmental pollution) can significantly modulate the associations of vitamin D with cardiovascular risk, including serum lipid levels [33, 46–50, 52–56]. For example, in the AFF group, there was a higher percentage of women compared with the two other groups, as well as a much higher percentage of the subjects of Hispanic origin, while in drivers was a higher percentage of the subjects of the non-Hispanic Black origin and in miners was a higher percentage of the subjects of the non-Hispanic white origin. Additionally, the percentage of those with vigorous physical activity, heavy alcohol consumption, and regular smoking was higher in the group of miners. Interestingly, miners exhibited the highest serum 25(OH)D levels, which is in accordance with some literature data [57]. One plausible explanation is the strenuous physical work undertaken by miners, leading to increased lipolysis and release of 25(OH)D from fat and muscle depots [58]. Studies have indicated that serum 25(OH)D levels tend to rise after intense physical activity, and individuals who engage in regular exercise tend to have higher 25(OH)D levels compared to sedentary individuals [53, 54]. Additionally, the high proportion of heavy alcohol consumption among miners may also contribute to their elevated vitamin D levels, as research suggested a positive correlation between alcohol consumption and serum 25(OH)D levels [55, 59]. Moreover, the AFF workers often operate in regions with harsh weather conditions, and they may encounter various chemical substances such as pesticides, fertilizers, and insecticides, which can contribute to a range of adverse health outcomes, as well as disturbed metabolism of vitamin D and lipids [16, 56]. It is important to note that prolonged sunlight exposure does not result in excessive production of vitamin D3, as the process of photo-conversion converts pre-vitamin D3 and vitamin D3 into their inactive metabolites [60]. Consequently, individuals with AFF occupations do not benefit from prolonged UVB exposure but are at an increased risk of developing skin cancer [61]. The exposure to various hazardous substances and prolonged presence in adverse environments can potentially impact the endocrine system and other physiological systems of individuals engaged in AFF, resulting in altered body metabolism compared to the general population [56]. This could serve as an additional potential explanation for the divergent associations observed between lipid profiles and serum 25(OH)D levels in the AFF group within the context of our study.

### Study limitations

This study has several limitations, each potentially impacting our findings. Firstly, the cross-sectional design hinders our ability to infer causality between vitamin D levels and lipid profiles. Secondly, there are significant seasonal variations in vitamin D levels between summer and winter [62], but the lack of specific timing for vitamin D measurements, due to NHANES’ data limitations, may have introduced variability related to seasonal fluctuations in vitamin D levels. This could lead to underestimation of vitamin D’s impact on lipid profiles. Thirdly, the indirect measurement of LDL-C levels using the Friedewald equation, particularly inappropriate for subjects with high TG levels, might have affected the accuracy of our lipid profile assessments. Fourth, the lack of matching for demographic and lifestyle factors among the control groups (traffic drivers and miners) and the AFF group could have introduced confounding variables. And our reliance on the broader occupational categories provided by the NHANES dataset inherently restricts our ability to distinguish between the varied roles and activities within each occupational field. For instance, the mining sector encompasses a range of job functions, from individuals operating cranes to those manually carrying heavy loads, as well as others engaged in more mobile roles. Such distinctions are crucial for a nuanced understanding of occupational impacts on health. This limitation underscores the necessity of more detailed occupational analyses in future research. Providing clear definitions of the included participants is essential for accurately interpreting the study’s findings and their implications. Fifth, due to the relatively small sample size in the AFF group, many of the correlation analyses were probably underpowered, especially when conducting subgroup analyses. Furthermore, the broad categorization of the AFF group in NHANES might have masked the nuances of different occupations within this category, possibly leading to a misinterpretation of the relationship between occupational exposure and vitamin D levels. Additionally, the absence of detailed dietary data, which could significantly influence vitamin D levels, is a notable limitation. Not accounting for this factor may have led to an incomplete understanding of the sources of variation in vitamin D levels among participants.

Given the non-linear nature of the found relationship, further research is necessary to determine the optimal vitamin D levels required for maintaining lipid balance across different populations. Such information would be valuable for developing relevant nutritional recommendations and clinical intervention strategies to enhance cardiovascular health and prevent cardiovascular diseases. However, despite these limitations, our results may provide a basis for implementing regular assessments of vitamin D status in AFF workers and considering supplementation in individuals with vitamin D deficiency or insufficiency to prevent an increase in blood lipids.

## Conclusions

In conclusion, our study highlights the intricate interplay between serum 25(OH)D levels and lipid profiles in AFF workers. These individuals exhibit unique non-linear associations of serum 25(OH)D levels with lipid profiles compared to other occupations. While further research is needed to explore underlying mechanisms and develop tailored interventions, maintaining adequate serum 25(OH)D levels may offer cardiovascular benefits to AFF workers. This understanding could contribute to improved cardiovascular health within this population.

## Supporting information

S1 Appendix Contains S1-S4 Tables and S1-S3 Figs. (DOC)
