# Supplementary material for: The unique association between serum 25-hydroxyvitamin D concentrations and blood lipid profiles in agriculture, forestry, and fishing occupations: Insights from NHANES 2001–2014
Source: PLoS One. 2024 Feb 27;19(2):e0297873. doi: 10.1371/journal.pone.0297873 (PMC10898752; doi:10.1371/journal.pone.0297873)
Supplement: S1 Appendix — (DOC) [file pone.0297873.s001.doc]

**S1 Table.** **Univariate analysis of Lipid profiles.**

|  | **Statistics** | **β (95%CI) P-value / OR (95%CI) P-value** | | | | | |
| --- | --- | --- | --- | --- | --- | --- | --- |
|  |  | **TG** | **TC** | **HDL** | **LDL** | **HDL/LDL** | **serum 25(OH)D** |
| Age (years) | 41.292 ± 13.903 | 0.010 (0.006, 0.014) <0.001*** | 0.017 (0.014, 0.019) <0.001*** | 0.001 (0.000, 0.002) 0.03* | 0.012 (0.009, 0.015) <0.001*** | -0.002 (-0.003, -0.001) <0.001*** | 0.004 (-0.046, 0.054) 0.87 |
| Serum glucose (mmol/L) | 5.648 ± 2.160 | 0.198 (0.174, 0.223) <0.001*** | 0.056 (0.040, 0.072) <0.001*** | -0.021 (-0.027, -0.016) <0.001*** | 0.025 (0.003, 0.047) 0.03* | -0.013 (-0.019, -0.007) <0.001*** | -0.754 (-1.076, -0.431) <0.001*** |
| BMI (kg/m^2^) | 28.827 ± 6.420 | 0.052 (0.044, 0.060) <0.001*** | 0.017 (0.011, 0.022) <0.001*** | -0.017 (-0.019, -0.015) <0.001*** | 0.015 (0.009, 0.022) <0.001*** | -0.009 (-0.011, -0.007) <0.001*** | -0.507 (-0.615, -0.399) <0.001*** |
| Working months | 142.296 ± 124.522 | 0.001 (0.000, 0.001) <0.01** | 0.001 (0.001, 0.002) <0.001*** | -0.000 (-0.000, 0.000) 0.74 | 0.001 (0.001, 0.001) <0.001*** | -0.000 (-0.000, -0.000) <0.001*** | 0.009 (0.001, 0.016) 0.02* |
| Arm circumference (cm) | 34.048 ± 4.710 | 0.058 (0.046, 0.069) <0.001*** | 0.017 (0.009, 0.025) <0.001*** | -0.024 (-0.027, -0.022) <0.001*** | 0.022 (0.012, 0.031) <0.001*** | -0.013 (-0.015, -0.010) <0.001*** | -0.528 (-0.677, -0.379) <0.001*** |
| Waist circumference (cm) | 99.406 ± 16.205 | 0.025 (0.021, 0.028) <0.001*** | 0.010 (0.008, 0.012) <0.001*** | -0.007 (-0.008, -0.007) <0.001*** | 0.008 (0.005, 0.011) <0.001*** | -0.004 (-0.005, -0.003) <0.001*** | -0.154 (-0.197, -0.110) <0.001*** |
| Creatinine (µmol/L) | 82.267 ± 31.639 | -0.000 (-0.002, 0.001) 0.59 | -0.001 (-0.002, -0.000) <0.05* | -0.000 (-0.001, -0.000) 0.01* | -0.001 (-0.002, 0.000) 0.24 | 0.001 (0.000, 0.001) <0.001*** | 0.030 (0.008, 0.053) 0.01** |
| Blood urea nitrogen (mmol/L) | 4.421 ± 1.628 | 0.094 (0.061, 0.127) <0.001*** | 0.023 (0.002, 0.045) 0.03* | -0.023 (-0.030, -0.015) <0.001*** | 0.024 (-0.003, 0.051) 0.09 | -0.012 (-0.020, -0.004) <0.01** | 2.027 (1.602, 2.451) <0.001*** |
| Serum uric acid (µmol/L) | 342.765 ± 80.120 | 0.003 (0.002, 0.003) <0.001*** | 0.001 (0.001, 0.002) <0.001*** | -0.001 (-0.001, -0.001) <0.001*** | 0.001 (0.001, 0.002) <0.001*** | -0.001 (-0.001, -0.000) <0.001*** | -0.003 (-0.012, 0.006) 0.47 |
| Glycohemoglobin (%) | 5.695 ± 1.153 | 0.300 (0.255, 0.346) <0.001*** | 0.124 (0.094, 0.154) <0.001*** | -0.038 (-0.049, -0.028) <0.001*** | 0.075 (0.038, 0.112) <0.001*** | -0.025 (-0.036, -0.014) <0.001*** | -1.943 (-2.545, -1.341) <0.001*** |
| Energy intake (kcal) | 2478.660 ± 1066.697 | -0.000 (-0.000, 0.000) 0.94 | -0.000 (-0.000, -0.000) <0.01** | -0.000 (-0.000, 0.000) 0.64 | -0.000 (-0.000, -0.000) <0.001*** | 0.000 (0.000, 0.000) <0.01** | 0.002 (0.001, 0.003) <0.001*** |
| Sex | | | | | | | |
| Males | 171 (4.343%) | 0 | 0 | 0 | 0 | 0 | 0 |
| Females | 3766 (95.657%) | -0.124 (-0.387, 0.140) 0.36 | -0.102 (-0.274, 0.071) 0.25 | -0.004 (-0.062, 0.054) 0.90 | -0.219 (-0.448, 0.010) 0.06 | 0.017 (-0.048, 0.083) 0.61 | 4.417 (1.004, 7.830) 0.01* |
| Race | | | | | | | |
| Hispanic | | | | | | | |
| Yes | 1477 (37.516%) | 0 | 0 | 0 | 0 | 0 | 0 |
| No | 2460 (62.484%) | -0.371 (-0.482, -0.261) <0.001*** | -0.131 (-0.203, -0.058) <0.001*** | 0.051 (0.027, 0.076) <0.001*** | -0.084 (-0.173, 0.005) 0.07 | 0.038 (0.013, 0.064) <0.01** | 4.818 (3.387, 6.248) <0.001*** |
| Non-Hispanic White | | | | | | | |
| Yes | 1494 (37.948%) | 0 | 0 | 0 | 0 | 0 | 0 |
| No | 2443 (62.052%) | -0.040 (-0.151, 0.071) 0.48 | 0.009 (-0.064, 0.081) 0.81 | 0.060 (0.036, 0.085) <0.001*** | 0.036 (-0.052, 0.124) 0.43 | 0.037 (0.012, 0.062) <0.01** | -18.309 (-19.625, -16.993) <0.001*** |
| Non-Hispanic Black | | | | | | | |
| Yes | 795 (20.193%) | 0 | 0 | 0 | 0 | 0 | 0 |
| No | 3142 (79.807%) | 0.633 (0.501, 0.766) <0.001*** | 0.204 (0.117, 0.292) <0.001*** | -0.162 (-0.191, -0.133) <0.001*** | 0.114 (0.008, 0.220) 0.04* | -0.111 (-0.141, -0.081) <0.001*** | 18.606 (16.971, 20.240) <0.001*** |
| Others | | | | | | | |
| Yes | 171 (4.343%) | 0 | 0 | 0 | 0 | 0 | 0 |
| No | 3766 (95.657%) | -0.124 (-0.387, 0.140) 0.36 | -0.102 (-0.274, 0.071) 0.25 | -0.004 (-0.062, 0.054) 0.90 | -0.219 (-0.448, 0.010) 0.06 | 0.017 (-0.048, 0.083) 0.61 | 4.417 (1.004, 7.830) 0.01* |
| Hypertension | | | | | | | |
| No | 2893 (74.047%) | 0 | 0 | 0 | 0 | 0 | 0 |
| Yes | 1014 (25.953%) | 0.338 (0.215, 0.461) <0.001*** | 0.118 (0.037, 0.199) <0.01** | -0.045 (-0.072, -0.018) <0.01** | -0.006 (-0.103, 0.092) 0.90 | -0.007 (-0.035, 0.021) 0.60 | -2.063 (-3.644, -0.482) 0.01* |
| Hyperlipidemia | | | | | | | |
| No | 1606 (64.576%) | 0 | 0 | 0 | 0 | 0 | 0 |
| Yes | 881 (35.424%) | 0.692 (0.556, 0.828) <0.001*** | 0.424 (0.333, 0.516) <0.001*** | -0.092 (-0.123, -0.062) <0.001*** | 0.234 (0.120, 0.349) <0.001*** | -0.060 (-0.094, -0.027) <0.001*** | 0.863 (-1.003, 2.729) 0.36 |
| Diabetes | | | | | | | |
| No | 3515 (89.372%) | 0 | 0 | 0 | 0 | 0 | 0 |
| Yes | 418 (10.628%) | 0.569 (0.396, 0.743) <0.001*** | -0.047 (-0.161, 0.068) 0.42 | -0.102 (-0.140, -0.064) <0.001*** | -0.165 (-0.301, -0.029) 0.02* | 0.004 (-0.035, 0.043) 0.82 | -4.284 (-6.540, -2.027) <0.001*** |
| Smoking status | | | | | | | |
| No | 1581 (42.580%) | 0 | 0 | 0 | 0 | 0 | 0 |
| Yes | 2132 (57.420%) | 0.178 (0.065, 0.292) <0.01** | 0.046 (-0.027, 0.119) 0.21 | -0.054 (-0.079, -0.029) <0.001*** | 0.024 (-0.065, 0.113) 0.60 | -0.018 (-0.044, 0.007) 0.16 | 1.869 (0.429, 3.310) 0.01* |
| Heavy alcohol | | | | | | | |
| No | 2287 (71.670%) | 0 | 0 | 0 | 0 | 0 | 0 |
| Yes | 904 (28.330%) | 0.230 (0.095, 0.365) <0.001*** | 0.034 (-0.053, 0.121) 0.44 | -0.008 (-0.037, 0.021) 0.60 | -0.096 (-0.204, 0.011) 0.08 | 0.033 (0.003, 0.064) 0.03* | 2.328 (0.633, 4.024) <0.01** |
| Vigorous work activity | | | | | | | |
| No | 2513 (63.847%) | 0 | 0 | 0 | 0 | 0 | 0 |
| Yes | 1423 (36.153%) | -0.131 (-0.243, -0.020) 0.02* | -0.144 (-0.217, -0.071) <0.001*** | -0.001 (-0.026, 0.024) 0.94 | -0.118 (-0.207, -0.029) <0.01** | 0.030 (0.004, 0.055) 0.02* | 6.552 (5.117, 7.986) <0.001*** |
| Education level | | | | | | | |
| Less than high school | 1621 (41.373%) | 0 | 0 | 0 | 0 | 0 | 0 |
| High school and above | 2297 (58.627%) | -0.170 (-0.279, -0.060) <0.01** | -0.059 (-0.130, 0.013) 0.11 | 0.024 (-0.000, 0.048) 0.05 | -0.016 (-0.104, 0.071) 0.72 | -0.000 (-0.025, 0.025) 0.97 | 3.871 (2.458, 5.284) <0.001*** |
| Marital status | | | | | | | |
| Married or with a partner | 2391 (62.477%) | 0 | 0 | 0 | 0 | 0 | 0 |
| Others | 1436 (37.523%) | -0.330 (-0.443, -0.217) <0.001*** | -0.181 (-0.255, -0.108) <0.001*** | 0.099 (0.075, 0.124) <0.001*** | -0.141 (-0.231, -0.052) <0.01** | 0.065 (0.040, 0.091) <0.001*** | -2.801 (-4.254, -1.349) <0.001*** |

* P ＜0.05, ** P ＜0.01, *** P ＜0.001.

**S2 Table. Univariate analysis of Lipid profiles in AFF occupations.**

|  | **Statistics** | **β (95%CI) P-value / OR (95%CI) P-value** | | | | | |
| --- | --- | --- | --- | --- | --- | --- | --- |
|  |  | **TG** | **TC** | **HDL** | **LDL** | **HDL/LDL** | **serum 25(OH)D** |
| Age (years) | 40.626 ± 14.927 | 0.014 (0.006, 0.023) <0.001*** | 0.020 (0.015, 0.026) <0.001*** | 0.001 (-0.001, 0.003) 0.37 | 0.015 (0.009, 0.022) <0.001*** | -0.002 (-0.004, -0.001) <0.01** | -0.080 (-0.192, 0.032) 0.16 |
| Serum glucose (mmol/L) | 5.654 ± 2.223 | 0.237 (0.184, 0.289) <0.001*** | 0.073 (0.033, 0.113) <0.001*** | -0.017 (-0.029, -0.005) <0.01** | 0.045 (-0.010, 0.099) 0.11 | -0.008 (-0.020, 0.004) 0.19 | -0.771 (-1.524, -0.017) <0.05* |
| BMI (kg/m^2^) | 28.579 ± 6.082 | 0.037 (0.018, 0.057) <0.001*** | 0.026 (0.011, 0.041) <0.001*** | -0.013 (-0.017, -0.008) <0.001*** | 0.021 (0.004, 0.039) 0.01* | -0.008 (-0.012, -0.005) <0.001*** | -0.625 (-0.900, -0.351) <0.001*** |
| Working months | 142.209 ± 125.723 | 0.001 (-0.000, 0.002) 0.21 | 0.001 (0.001, 0.002) <0.01** | -0.000 (-0.000, 0.000) 0.37 | 0.002 (0.001, 0.003) <0.01** | -0.000 (-0.000, -0.000) 0.01* | -0.015 (-0.031, 0.001) 0.06 |
| Arm circumference (cm) | 33.265 ± 4.490 | 0.036 (0.010, 0.063) <0.01** | 0.019 (-0.002, 0.039) 0.07 | -0.021 (-0.027, -0.015) <0.001*** | 0.020 (-0.003, 0.044) 0.09 | -0.012 (-0.017, -0.007) <0.001*** | -0.335 (-0.713, 0.042) 0.08 |
| Waist circumference (cm) | 98.067 ± 15.327 | 0.019 (0.012, 0.027) <0.001*** | 0.013 (0.008, 0.019) <0.001*** | -0.006 (-0.007, -0.004) <0.001*** | 0.012 (0.005, 0.019) <0.001*** | -0.004 (-0.005, -0.002) <0.001*** | -0.214 (-0.325, -0.103) <0.001*** |
| Creatinine (µmol/L) | 74.549 ± 18.232 | -0.003 (-0.010, 0.003) 0.32 | -0.005 (-0.010, -0.000) <0.05* | -0.002 (-0.004, -0.001) <0.01** | 0.004 (-0.001, 0.010) 0.14 | -0.002 (-0.003, -0.001) <0.01** | 0.177 (0.086, 0.268) <0.001*** |
| Blood urea nitrogen (mmol/L) | 4.543 ± 1.748 | 0.026 (-0.045, 0.097) 0.47 | -0.000 (-0.052, 0.051) 0.99 | -0.018 (-0.033, -0.003) 0.02* | 0.039 (-0.027, 0.105) 0.25 | -0.013 (-0.027, 0.002) 0.09 | 1.917 (0.967, 2.866) <0.001*** |
| Serum uric acid (µmol/L) | 326.364 ± 83.649 | 0.001 (-0.001, 0.002) 0.22 | 0.000 (-0.001, 0.001) 0.61 | -0.001 (-0.001, -0.000) <0.001*** | 0.001 (-0.001, 0.002) 0.37 | -0.000 (-0.001, -0.000) <0.001*** | 0.013 (-0.007, 0.033) 0.19 |
| Glycohemoglobin (%) | 5.703 ± 1.217 | 0.373 (0.276, 0.470) <0.001*** | 0.180 (0.108, 0.252) <0.001*** | -0.018 (-0.039, 0.004) 0.11 | 0.112 (0.023, 0.201) 0.01* | -0.017 (-0.036, 0.002) 0.09 | -1.959 (-3.322, -0.596) <0.01** |
| Energy intake (kcal) | 2315.648 ± 1040.524 | 0.000 (-0.000, 0.000) 0.27 | -0.000 (-0.000, 0.000) 0.93 | -0.000 (-0.000, -0.000) 0.02* | -0.000 (-0.000, 0.000) 0.84 | -0.000 (-0.000, 0.000) 0.20 | 0.003 (0.001, 0.004) <0.01** |
| Sex | | | | | | | |
| Males | 453 (72.480%) | 0 | 0 | 0 | 0 | 0 | 0 |
| Females | 172 (27.520%) | -0.143 (-0.419, 0.134) 0.31 | 0.169 (-0.030, 0.369) 0.10 | 0.189 (0.131, 0.246) <0.001*** | -0.023 (-0.257, 0.211) 0.85 | 0.084 (0.034, 0.134) <0.01** | -5.357 (-9.069, -1.644) <0.01** |
| Race | | | | | | | |
| Hispanic | | | | | | | |
| Yes | 377 (60.320%) | 0 | 0 | 0 | 0 | 0 | 0 |
| No | 248 (39.680%) | -0.305 (-0.556, -0.054) 0.02* | -0.077 (-0.260, 0.105) 0.41 | 0.052 (-0.002, 0.106) 0.06 | -0.154 (-0.367, 0.060) 0.16 | 0.047 (0.000, 0.093) <0.05* | 14.307 (11.086, 17.527) <0.001*** |
| Non-Hispanic White | | | | | | | |
| Yes | 192 (30.720%) | 0 | 0 | 0 | 0 | 0 | 0 |
| No | 433 (69.280%) | 0.253 (-0.013, 0.520) 0.06 | 0.076 (-0.117, 0.270) 0.44 | -0.023 (-0.081, 0.034) 0.43 | 0.290 (0.069, 0.511) 0.01* | -0.058 (-0.106, -0.010) 0.02* | -20.006 (-23.264, -16.748) <0.001*** |
| Non-Hispanic Black | | | | | | | |
| Yes | 38 (6.080%) | 0 | 0 | 0 | 0 | 0 | 0 |
| No | 587 (93.920%) | 0.685 (0.167, 1.204) <0.01** | 0.186 (-0.192, 0.563) 0.34 | -0.138 (-0.250, -0.027) 0.02* | -0.326 (-0.749, 0.098) 0.13 | 0.022 (-0.071, 0.114) 0.65 | 13.523 (6.622, 20.425) <0.001*** |
| Others | | | | | | | |
| Yes | 18 (2.880%) | 0 | 0 | 0 | 0 | 0 | 0 |
| No | 607 (97.120%) | -0.733 (-1.487, 0.021) 0.06 | -0.290 (-0.822, 0.243) 0.29 | 0.011 (-0.148, 0.169) 0.89 | -0.441 (-1.248, 0.365) 0.28 | 0.016 (-0.160, 0.191) 0.86 | 2.192 (-7.785, 12.168) 0.67 |
| Hypertension | | | | | | | |
| No | 485 (80.298%) | 0 | 0 | 0 | 0 | 0 | 0 |
| Yes | 119 (19.702%) | 0.376 (0.061, 0.690) 0.02* | 0.275 (0.046, 0.503) 0.02* | 0.000 (-0.068, 0.068) 0.10 | 0.167 (-0.097, 0.430) 0.22 | 0.003 (-0.054, 0.060) 0.91 | -1.246 (-5.538, 3.047) 0.57 |
| Hyperlipidemia | | | | | | | |
| No | 196 (64.474%) | 0 | 0 | 0 | 0 | 0 | 0 |
| Yes | 108 (35.526%) | 0.613 (0.235, 0.992) <0.01** | 0.799 (0.537, 1.061) <0.001*** | 0.018 (-0.057, 0.093) 0.64 | 0.255 (-0.081, 0.590) 0.14 | -0.018 (-0.078, 0.043) 0.56 | 2.458 (-2.872, 7.788) 0.37 |
| Diabetes | | | | | | | |
| No | 568 (91.026%) | 0 | 0 | 0 | 0 | 0 | 0 |
| Yes | 56 (8.974%) | 0.198 (-0.232, 0.629) 0.37 | 0.120 (-0.192, 0.432) 0.45 | 0.030 (-0.063, 0.123) 0.53 | 0.065 (-0.259, 0.388) 0.70 | 0.004 (-0.066, 0.075) 0.91 | -4.009 (-9.836, 1.818) 0.18 |
| Smoking status | | | | | | | |
| No | 317 (56.306%) | 0 | 0 | 0 | 0 | 0 | 0 |
| Yes | 246 (43.694%) | 0.330 (0.063, 0.598) 0.02* | 0.270 (0.084, 0.456) <0.01** | -0.060 (-0.118, -0.003) 0.04* | 0.352 (0.134, 0.569) <0.01** | -0.074 (-0.121, -0.027) <0.01** | 0.189 (-3.334, 3.713) 0.92 |
| Heavy alcohol | | | | | | | |
| No | 333 (75.682%) | 0 | 0 | 0 | 0 | 0 | 0 |
| Yes | 107 (24.318%) | -0.022 (-0.377, 0.332) 0.90 | 0.065 (-0.183, 0.313) 0.61 | -0.005 (-0.079, 0.069) 0.89 | 0.028 (-0.265, 0.322) 0.85 | -0.004 (-0.066, 0.058) 0.90 | -1.868 (-6.515, 2.778) 0.43 |
| Vigorous work activity | | | | | | | |
| No | 427 (68.320%) | 0 | 0 | 0 | 0 | 0 | 0 |
| Yes | 198 (31.680%) | -0.177 (-0.442, 0.088) 0.19 | -0.241 (-0.432, -0.050) 0.01* | -0.035 (-0.092, 0.022) 0.22 | -0.279 (-0.507, -0.051) 0.02* | 0.049 (-0.001, 0.099) 0.05 | 8.998 (5.481, 12.514) <0.001*** |
| Education level | | | | | | | |
| Less than high school | 373 (60.848%) | 0 | 0 | 0 | 0 | 0 | 0 |
| High school and above | 240 (39.152%) | -0.050 (-0.307, 0.206) 0.70 | -0.054 (-0.239, 0.130) 0.56 | -0.005 (-0.060, 0.050) 0.85 | -0.116 (-0.336, 0.105) 0.31 | 0.037 (-0.011, 0.084) 0.13 | 13.092 (9.783, 16.401) <0.001*** |
| Marital status | | | | | | | |
| Married or with a partner | 405 (66.285%) | 0 | 0 | 0 | 0 | 0 | 0 |
| Others | 206 (33.715%) | -0.427 (-0.690, -0.164) <0.01** | -0.295 (-0.484, -0.106) <0.01** | 0.004 (-0.052, 0.061) 0.88 | -0.082 (-0.304, 0.140) 0.50 | 0.016 (-0.031, 0.063) 0.51 | -3.701 (-7.276, -0.127) 0.04* |

* P ＜0.05, ** P ＜0.01, *** P ＜0.001

**S3 Table. The stratified analyses of the associations of serum 25(OH)D concentrations with lipid profiles in AFF occupations (without BMI as a covariate)**

|  | **Outcomes:** (β (95% CI) P-value) | | | | |
| --- | --- | --- | --- | --- | --- |
|  | **TG (mmol/L)** | **TC (mmol/L)** | **HDL-C (mmol/L)** | **LDL-C (mmol/L)** | **HDL-C/LDL-C** |
| **Total** | -0.002 (-0.010, 0.007) 0.68 | 0.000 (-0.006, 0.006) 0.99 | 0.002 (0.000, 0.004) 0.03* | 0.000 (-0.006, 0.006) 0.97 | 0.001 (-0.000, 0.002) 0.08 |
| **Serum 25(OH)D group (nmol/L)** | | | | | |
| < 50 | 0.026 (-0.008, 0.060) 0.13 | 0.018 (-0.008, 0.045) 0.18 | -0.004 (-0.012, 0.004) 0.33 | -0.011 (-0.045, 0.023) 0.53 | 0.000 (-0.006, 0.006) 0.94 |
| ≥ 50 | -0.011 (-0.023, 0.001) 0.07 | -0.004 (-0.012, 0.005) 0.40 | 0.004 (0.002, 0.006) <0.001*** | 0.002 (-0.007, 0.010) 0.71 | 0.002 (0.000, 0.004) 0.04* |
| **Age (years)** | | | | | |
| < 40 | -0.001 (-0.015, 0.013) 0.88 | 0.002 (-0.008, 0.013) 0.69 | 0.002 (-0.001, 0.005) 0.11 | -0.005 (-0.018, 0.007) 0.39 | 0.002 (-0.001, 0.005) 0.14 |
| ≥ 40 | -0.002 (-0.012, 0.008) 0.71 | -0.001 (-0.008, 0.006) 0.84 | 0.002 (-0.000, 0.004) 0.09 | 0.001 (-0.006, 0.009) 0.72 | 0.001 (-0.000, 0.002) 0.11 |
| **Sex** | | | | | |
| Males | 0.001 (-0.010, 0.011) 0.87 | 0.000 (-0.007, 0.007) 0.97 | 0.001 (-0.001, 0.003) 0.54 | 0.001 (-0.007, 0.009) 0.72 | 0.001 (-0.001, 0.003) 0.25 |
| Females | -0.008 (-0.020, 0.004) 0.21 | -0.001 (-0.012, 0.009) 0.80 | 0.004 (0.001, 0.007) 0.01* | -0.003 (-0.014, 0.009) 0.67 | 0.002 (-0.001, 0.004) 0.19 |
| **BMI** **(kg/m^2^)** | | | | | |
| < 30 | -0.003 (-0.013, 0.007) 0.55 | 0.003 (-0.004, 0.010) 0.41 | 0.003 (0.001, 0.005) <0.01** | 0.002 (-0.005, 0.009) 0.61 | 0.001 (-0.000, 0.003) 0.12 |
| ≥ 30 | 0.005 (-0.009, 0.018) 0.49 | -0.003 (-0.015, 0.008) 0.59 | -0.002 (-0.005, 0.000) 0.09 | 0.001 (-0.014, 0.017) 0.85 | -0.001 (-0.003, 0.002) 0.56 |
| **Vigorous work activity** | | | | | |
| Yes | -0.003 (-0.016, 0.010) 0.65 | -0.004 (-0.015, 0.007) 0.45 | 0.003 (-0.000, 0.006) 0.07 | -0.001 (-0.013, 0.010) 0.80 | 0.002 (-0.001, 0.005) 0.23 |
| No | -0.001 (-0.011, 0.010) 0.88 | 0.002 (-0.005, 0.009) 0.66 | 0.001 (-0.001, 0.003) 0.20 | 0.000 (-0.008, 0.008) 0.94 | 0.001 (-0.001, 0.002) 0.33 |
| **Working months** | | | | | |
| < 200 | -0.001 (-0.018, 0.015) 0.88 | -0.004 (-0.016, 0.007) 0.48 | 0.001 (-0.002, 0.005) 0.39 | -0.007 (-0.020, 0.007) 0.32 | 0.002 (-0.001, 0.005) 0.24 |
| ≥ 200 | 0.003 (-0.018, 0.024) 0.77 | 0.000 (-0.016, 0.016) 0.96 | -0.000 (-0.005, 0.004) 0.83 | 0.006 (-0.014, 0.027) 0.54 | -0.000 (-0.003, 0.002) 0.76 |
| **Heavy alcohol** | | | | | |
| Yes | 0.002 (-0.015, 0.020) 0.79 | 0.006 (-0.006, 0.018) 0.33 | 0.003 (-0.001, 0.006) 0.18 | 0.001 (-0.012, 0.013) 0.94 | 0.001 (-0.002, 0.004) 0.36 |
| No | -0.003 (-0.012, 0.007) 0.60 | -0.001 (-0.008, 0.005) 0.70 | 0.002 (-0.000, 0.004) 0.05 | -0.000 (-0.008, 0.007) 0.95 | 0.002 (0.000, 0.003) 0.04* |

Sex, age, each particular race, smoking, heavy alcohol, diabetes, hypertension, vigorous physical activity, education level, and marital status were adjusted except the variable itself. (continuous age was adjusted in the age subgroup) * P <0.05, ** P <0.01, *** P <0.001

**S4 Table.** **Threshold effect analysis of serum 25(OH)D concentrations on lipid profiles (without BMI as a covariate)**

|  | **Adjusted HR (95% CI) P-value** |
| --- | --- |
| **TG** |  |
| Fitting by the standard linear model | -0.002 (-0.010, 0.007) 0.68 |
| Fitting by the two-piecewise linear model |  |
| Infection point | 64.1 |
| serum 25(OH)D < Infection point | 0.015 (-0.001, 0.030) 0.06 |
| serum 25(OH)D≥ Infection point | -0.015 (-0.029, -0.002) 0.03* |
| P for Log-likelihood ratio | 0.01* |
| **TC** |  |
| Fitting by the standard linear model | -0.000 (-0.006, 0.006) 0.94 |
| Fitting by the two-piecewise linear model |  |
| Infection point | 49.5 |
| serum 25(OH)D < Infection point | 0.017 (-0.001, 0.036) 0.07 |
| serum 25(OH)D ≥ Infection point | -0.005 (-0.013, 0.003) 0.19 |
| P for Log-likelihood ratio0 | < 0.05* |
| **HDL-C** |  |
| Fitting by the standard linear model | 0.002 (0.000, 0.004) 0.03* |
| Fitting by the two-piecewise linear model |  |
| Infection point | 49.2 |
| serum 25(OH)D < Infection point | -0.005 (-0.010, 0.001) 0.09 |
| serum 25(OH)D ≥ Infection point | 0.004 (0.001, 0.006) < 0.01* |
| P for Log-likelihood ratio0 | 0.01* |
| **LDL-C** |  |
| Fitting by the standard linear model | 0.000 (-0.006, 0.006) 0.97 |
| Fitting by the two-piecewise linear model |  |
| Infection point | 58.1 |
| serum 25(OH)D < Infection point | -0.004 (-0.019, 0.011) 0.61 |
| serum 25(OH)D ≥ Infection point | 0.002 (-0.007, 0.010) 0.68 |
| P for Log-likelihood ratio0 | 0.55 |
| **HDL-C/LDL-C** |  |
| Fitting by the standard linear model | 0.001 (-0.000, 0.002) 0.08 |
| Fitting by the two-piecewise linear model |  |
| Infection point | 65.8 |
| serum 25(OH)D< Infection point | -0.000 (-0.003, 0.002) 0.85 |
| serum 25(OH)D ≥ Infection point | 0.002 (0.000, 0.004) 0.03* |
| P for Log-likelihood ratio0 | 0.17 |

Sex, age, each particular race, smoking, heavy alcohol, diabetes, hypertension, vigorous physical activity, education level, and marital status were adjusted. * P < 0.05


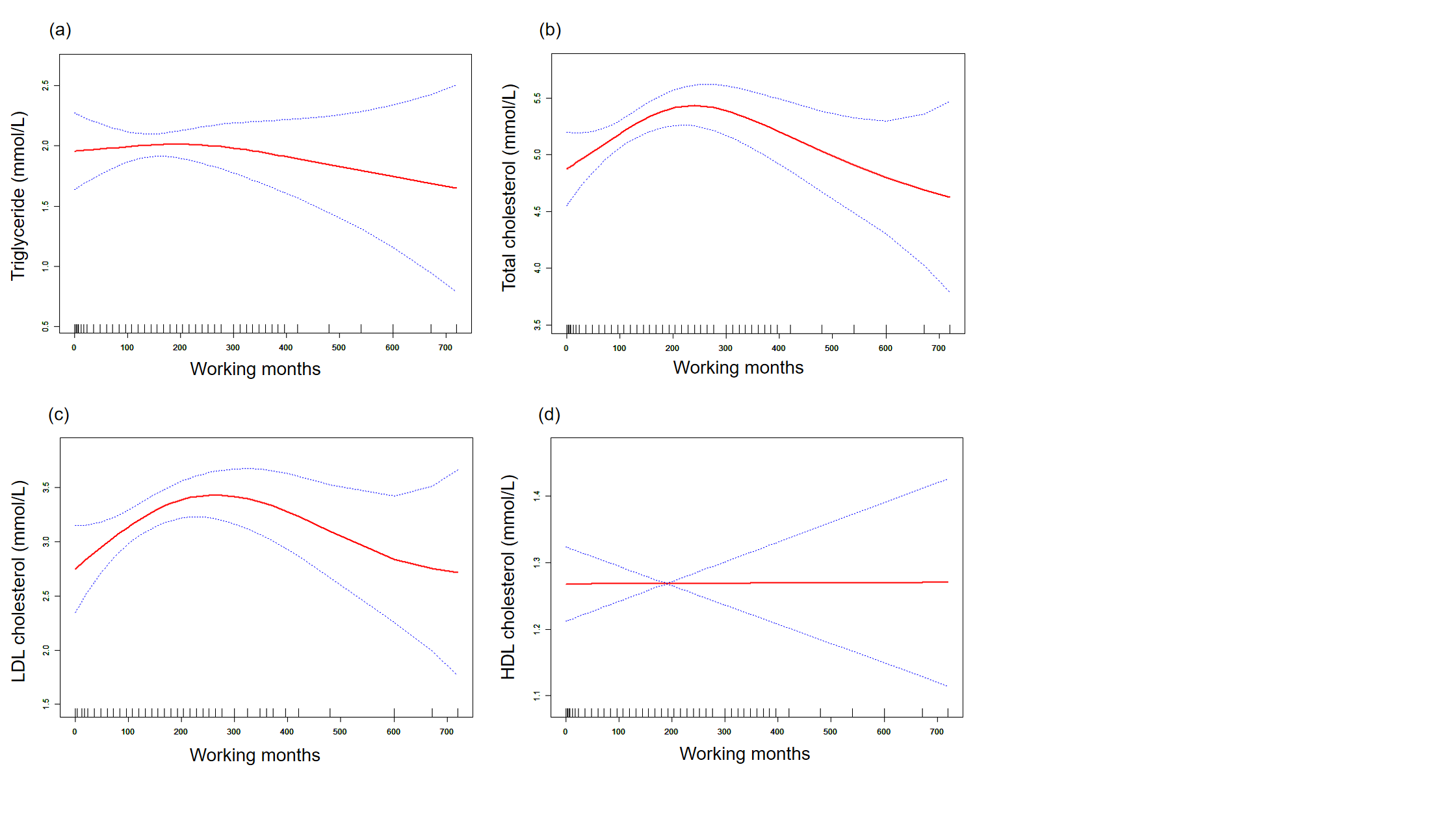


**S1 Fig. The associations between lipids and working months of AFF.** **(a,** **b, c, d)** Associations of working months with triglycerides (mmol/L), total cholesterol (mmol/L), LDL cholesterol (mmol/L) and HDL cholesterol (mmol/L) of AFF.


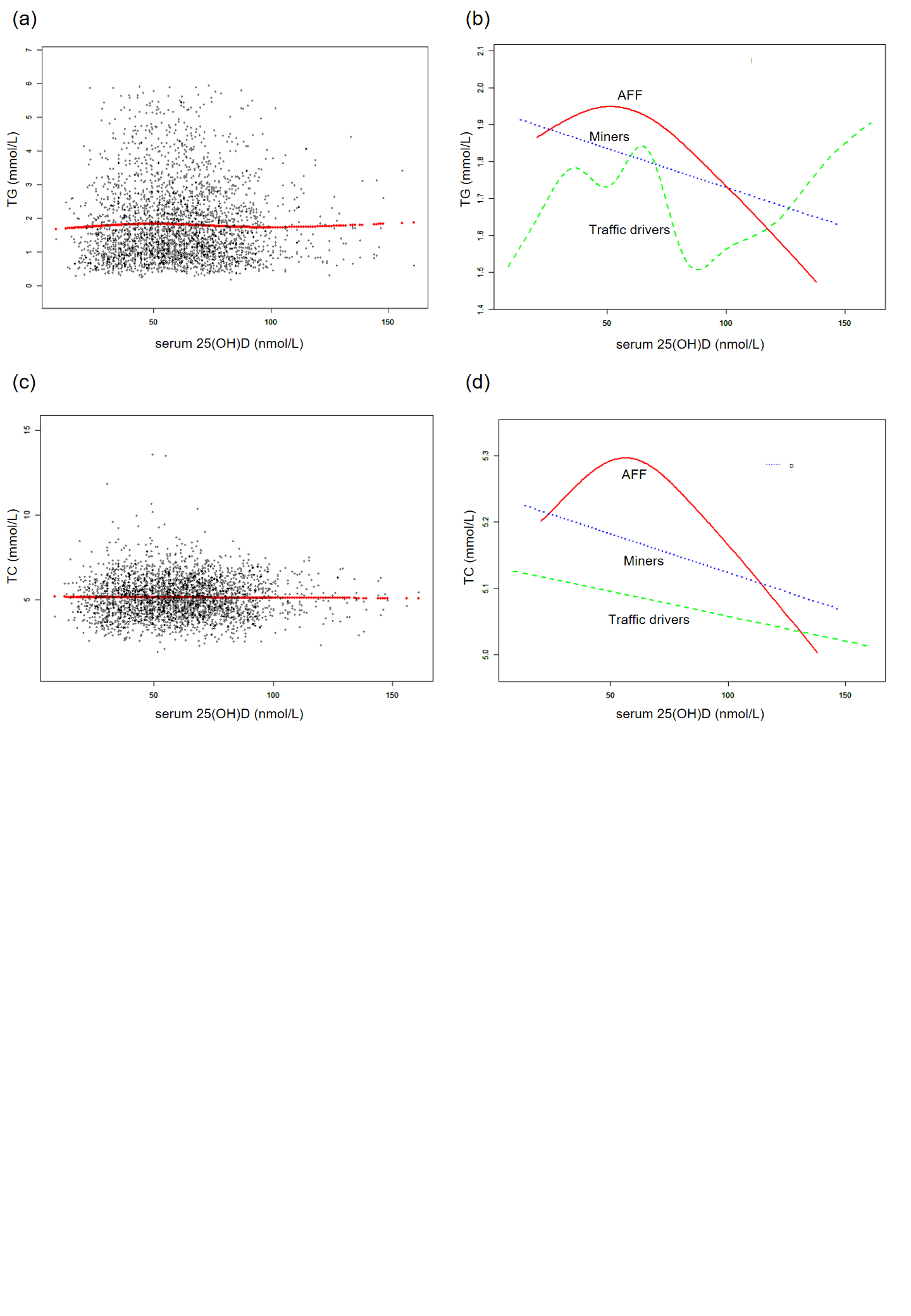


**S2 Fig. The associations between serum 25(OH)D concentrations and triglycerides, total cholesterol. (a, c)** Each black point represents a sample and the red line represents the general trend of these samples. **(b, d)** Associations of serum 25(OH)D concentrations with triglycerides, total cholesterol stratified by occupations. Sex, age, each particular race, smoking, heavy alcohol, diabetes, hypertension, vigorous physical activity, education level, and marital status were adjusted.


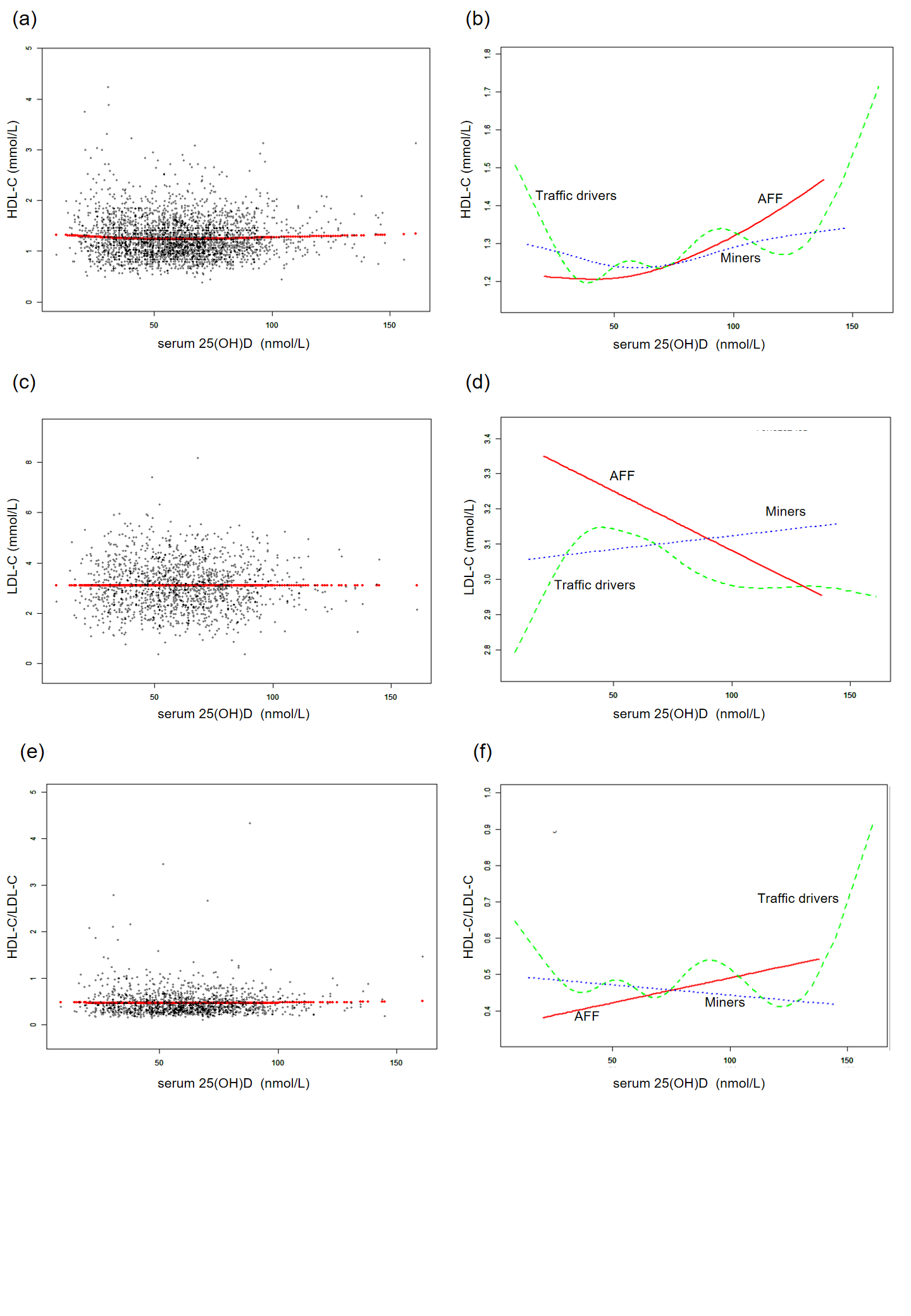


**S3 Fig. The associations between serum 25(OH)D concentrations and LDL cholesterol, LDL cholesterol, HDL-C/LDL-C. (a, c, e)** Each black point represents a sample and the red line represents the general trend of these samples. **(b, d, f)** Associations of serum 25(OH)D concentrations with LDL cholesterol, HDL cholesterol and HDL-C/LDL/C stratified by occupations. Sex, age, each particular race, smoking, heavy alcohol, diabetes, hypertension, vigorous physical activity, education level, and marital status were adjusted.
